# Leveraging Patient-Reported Outcome Measures for Optimal Dose Selection in Early Phase Cancer Trials

**DOI:** 10.2196/64611

**Published:** 2025-02-28

**Authors:** Bill Byrom, Anthony Everhart, Paul Cordero, Chris Garratt, Tim Meyer

**Affiliations:** 1 Science and Medicine, Signant Health, 785 Arbor Way, Blue Bell, PA, 19422, United States, 44 20 4526 1340; 2 Patient Informed Development & Health Value Translation, Sanofi Pharmaceuticals, Reading, United Kingdom; 3 New Medicines Development, Orion Pharma, Nottingham, United Kingdom; 4 Research Department of Oncology, University College London, London, United Kingdom

**Keywords:** clinical trials, early phase, dose finding, patient-reported outcome, PRO, electronic patient-reported outcome, ePRO, PRO-CTCAE, adverse events, tolerability, optimal dose, cancer trials, dose toxicity, oncology, drug development, electronic collection, dose level, pharmacodynamic, cytotoxic chemotherapy drugs, cytotoxic, chemotherapy drug, life-threatening disease, Common Terminology Criteria for Adverse Events

## Abstract

While patient-reported outcome measures are regularly incorporated into phase 3 clinical trials, they have been infrequently used in early phase trials. However, the patient’s perspective is vital to fully understanding dose toxicity and selecting an optimal dose. This viewpoint paper reviews the rationale for and practical approach to collecting patient-reported outcome data in early phase oncology drug development and the rationale for electronic collection.

## Dose Finding in Oncology Drug Development

**Figure 1.**
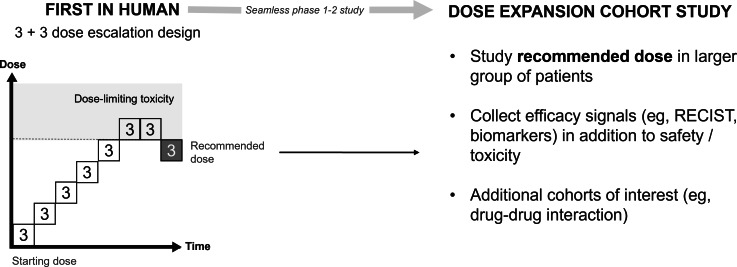
Traditional early phase oncology dose-finding studies. RECIST: Response Evaluation Criteria in Solid Tumors.

Traditional dose findings in oncology clinical drug development have focused on determining the maximum tolerated dose, assuming that the efficacy-dose relationship follows a steep monotonic increasing curve. By this assumption, the recommended dose is defined as the highest dose in which dose-limiting toxicity is not observed. Typical early phase programs (Figure 1) often use a 3 + 3 dose escalation design in which subsequent cohorts of 3 patients are studied, each receiving a higher dose than the last. Dose levels often follow a modified Fibonacci sequence whereby dose increments become smaller as the dose increases [1]. When dose-limiting toxicity is observed in at least one patient, the dose level is repeated in a further cohort of 3 patients, and if dose-limiting toxicity is observed again, further escalation stops, identifying the previous dose as the recommended maximum tolerated dose to take forward. Further study of the recommended dose is achieved, often using a seamless phase 1-2 design, by recruiting an additional larger group of patients into a dose expansion study. The primary end point in an expansion cohort is usually to determine efficacy, most frequently according to the radiological response rate. Additionally, further safety data is gathered, and pharmacodynamic markers may also be developed.

**Figure 2.**
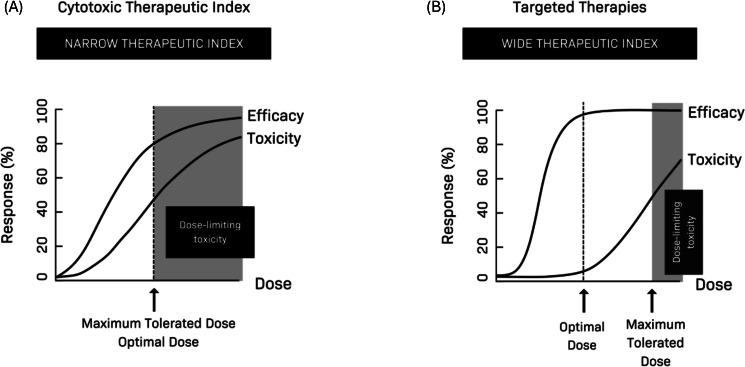
Dose-response relationships and optimal dose selection for cytotoxic chemotherapy drugs and targeted therapies.

This approach has been acceptable for cytotoxic chemotherapy drugs due to their steep dose-response relationships, their limited drug target specificity, and the willingness of patients to trade off substantial toxicity to treat serious, life-threatening diseases [2]. However, it may lead to the recommendation of higher doses and a suboptimal tolerability profile when used in dose finding for modern, more targeted oncology drugs such as kinase inhibitors and monoclonal antibodies (Figure 2). In these cases, the wider therapeutic index means that a range of doses may show relevant efficacy, and doses below the maximum tolerated dose may have similar efficacy with reduced toxicity [3]. This can be particularly important because targeted therapies are often taken for much longer periods, during which lower grade, persistent symptomatic toxicities can present a greater challenge to patients [2]. Dose finding limitations have been illustrated in 26% of Food and Drug Administration (FDA)–approved kinase inhibitors (2001‐2015) requiring postmarketing requirements/commitments to study alternative doses [4].

Better characterizing dose response to optimize dose finding has been underlined by the FDA’s Project Optimus, which aims to reform how doses are selected in oncology clinical trials, with a particular focus on maximizing efficacy and optimizing safety and tolerability [5]. This led to their subsequent guidance on dose optimization for new cancer treatments [2]. Studying more dose levels in the dose expansion study may be one approach to enable this and may better enable characterization of the dose-response relationship, albeit qualitatively given the likely small cohort sizes.

## Understanding Tolerability

Tolerability is defined in good clinical practice as “the degree to which overt adverse effects can be tolerated by the subject” [6]. In oncology, assessment of tolerability typically comprises clinician-reported treatment-related adverse events (AEs) using, for example, the Common Terminology Criteria for Adverse Events (CTCAE) [7], along with other data such as dose modifications, discontinuations and interruptions, safety biomarkers, hospitalization, and death [8]. However, these tools and data fail to fully account for the patient’s perspective or to fully measure the impact of AEs on the patient’s activities and quality of life. Many studies comparing physician and patient reports of treatment-related AEs have consistently shown underreporting and reduced severity rating in physician interpretations compared to patient reports [9-16]. This represents a challenge for drug developers in accurately quantifying the dose-toxicity relationship and limits the ability to define optimal doses, leading to a greater risk of exposing greater numbers of patients to doses that are too high, potentially resulting in increased discontinuation and a less favorable safety profile.

For example, measuring the frequency of individual AEs reported by early phase patients using the full Patient-Reported Outcomes version of the CTCAE (PRO-CTCAE) item bank [17], Veitch and colleagues [9] evaluated the associated frequency of reporting of the same AEs by physicians using the CTCAE. They found that all 50 AEs reported by at least 10% of patients undergoing cancer treatment (n=243) were consistently underreported by physicians using the CTCAE, in some cases markedly. For example, 9 AEs were identified at least 50 times less frequently by physicians: decreased libido (31.4% vs 0.1%), palpitations (14.7% vs 0.1%), wheezing (14.5% vs 0.2%), voice alteration (14.1% vs 0.2%), hiccups (13.9% vs 0.1%), hyperhidrosis (23.9% vs 0.4%), vaginal dryness (11.0% vs 0.1%), nail ridging (10.0% vs 0.2%), and urinary incontinence (10.0% vs 0.2%). Further, 19 CTCAE items were reported 1% or less of the time by physicians, compared to 10%-31.4% by patients.

A further study in 1933 patients with a variety of oncology conditions treated in various routine care settings reported an underestimation of AE severity by clinicians in comparison to patient reports [10]. The frequency of symptoms assessed as moderate or severe by patients and physicians, respectively, were pain (67% vs 47%), fatigue (71% vs 54%), generalized weakness (65% vs 47%), anorexia (47% vs 25%), depression (31% vs 17%), constipation (45% vs 30%), poor sleep (32% vs 21%), dyspnea (30% vs 16%), nausea (27% vs 14%), vomiting (14% vs 6%), and diarrhea (14 vs 6%). While this study was not conducted in an early phase setting, it is likely that the discordance in clinician and patient assessments, consistent with Veitch et al [9], would be similarly reflected in early phase research.

These examples demonstrate that physician assessment of patient AEs may be both incomplete and underestimated in comparison to the patient perspective. Reasons for this may include patient difficulties in spontaneously raising or describing AEs, patient fears of delay or discontinuation of treatment options in which they have high expectations of positive results, introduction of clinician subjectivity, and time constraints and practical limitations with current physician tools.

While valuable in addressing the underreporting and lower scoring of AE severity by physicians, the PRO-CTCAE alone fails to assess the cumulative impact of the AE profile and the effects on functioning and quality of life. The cumulative impact may be especially important in newer treatments taken for sustained periods, where multiple, concurrent, low-grade but persistent AEs may together represent an intolerable burden for the patient. As we describe later, supplementing the rating of individual AEs with an overall single-item measure of the cumulative impact of AEs (eg, using the Functional Assessment of Cancer Therapy–Item GP5 [FACT GP5] [18] or item 168 from the European Organisation for Research and Treatment of Cancer [EORTC] item library [19]) and the adverse impact on patient physical function and role function (eg, measured using the associated subscales of the EORTC Quality of Life Questionnaire—Core Questionnaire [EORTC QLQ-C30] [20]) provides a valuable assessment of the impact of the AE profile experienced.

## Using Patient-Reported Outcomes in Early Phase Oncology Trials

### Overview

While the use of patient-reported outcome measures (PROMs) is increasingly incorporated into phase 3 clinical trials, and regulatory recommendations on measurement strategy have been recently published by the FDA [21], there is little use of PROMs in early phase trials [22]. Barriers to adoption in early phase oncology studies include a lack of guidance regarding PROM selection, concerns relating to dealing with missing patient-reported outcome (PRO) data, overburdening site staff and patients, handling patient and data queries [23], and low power associated with small sample sizes. Nonetheless, the patient perspective is a vital element of fully understanding dose toxicity and selecting an optimal dose for later phase development.

### Adverse Events

In later phase trials, there is typically enough understanding of the AE profile of the investigational treatment to enable a reliable preselection of items for measurement (eg, using a small subset of PRO-CTCAE items). The same is not true for first in human and other early phase trials. Preclinical data may provide some signals to drive thinking, but these are unlikely to be robust and comprehensive, and while the AE evidence from other drugs with the same mechanism of action may be available and relevant, this is not always the case. The full PRO-CTCAE instrument contains 124 items across 78 distinct terms [17], and this is impractical to use in a full list format for regular ongoing measurement.

Janse van Rensburg and colleagues [24] used a statistical approach to develop a reduced list of PRO-CTCAE items considered most likely to occur in a phase 1 population using the same dataset reported by Veitch et al [9]. Using that dataset, they eliminated AEs recorded less than 5% of the time; those recorded as “mild” severity by at least 75% of patients; and AEs associated with interference scores of “not at all,” frequency scores of “never,” or amount scores of “not at all” by at least 80% of patients. Finally, terms with the lowest internal reliability within each organ system domain, as measured using Cronbach α, were also eliminated. With further refinements from physician perspectives, this led to a tailored PRO-CTCAE survey consisting of 58 items assessing 30 terms. While a useful and interesting approach, the generalizability of this reduced survey may be limited by the relatively small sample size, the limited set of treatments and tumor types represented, and the risk that using historical data may miss important aspects of tolerability for new targeted therapies.

When selecting a reduced set of predefined items, it is helpful, as PRO-CTCAE recommends and allows, to include a free-text item to capture other important AEs not listed [25] and to use this information to allow the item list to adapt with the emerging understanding gained through continued study.

**Figure 3.**
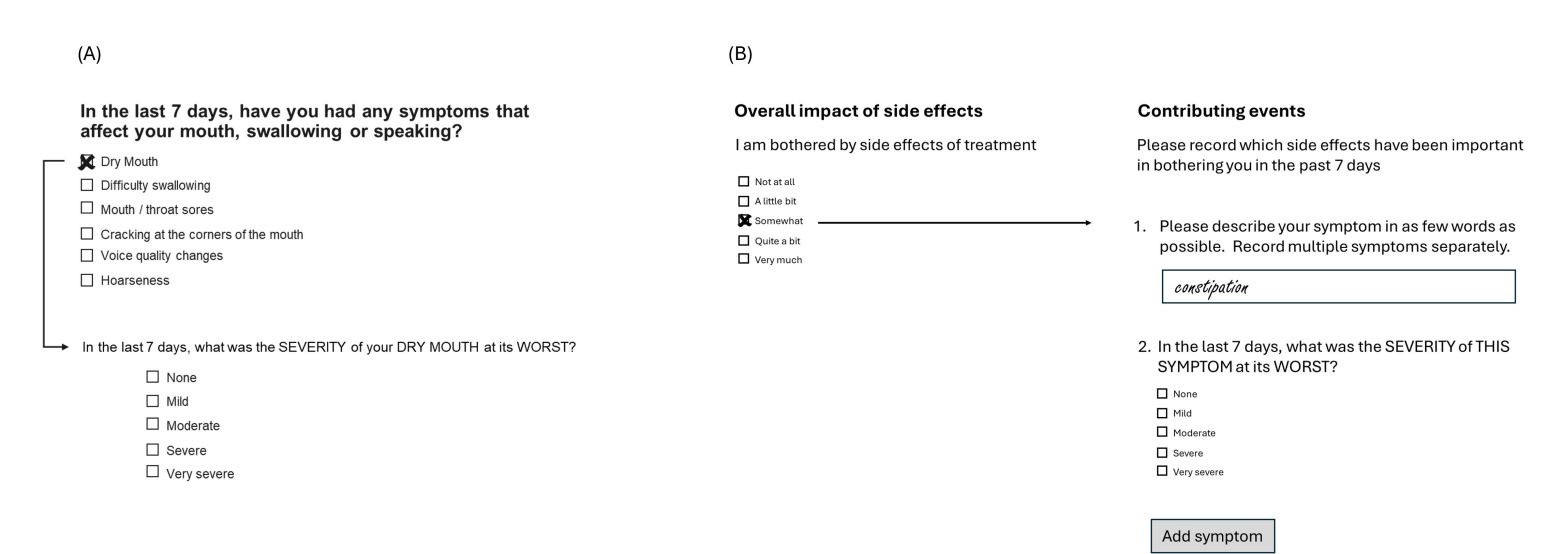
Approaches to simplify adverse event capture and scoring (A) using organ system grouping and (B) collecting the most bothersome adverse events associated with overall impact score.

Because the PRO-CTCAE items are grouped by organ system domains, it is possible to optimize the completion of the full item list compared to an individual symptom-by-symptom approach (Figure 3A). Alternatively, a free-text approach asking patients to list and rate the AEs that contribute most to their overall impact rating (scored using the FACT GP5 item, for example) might provide a less burdensome approach (Figure 3B). Leveraging an electronic PRO (ePRO) solution, using a smartphone app, for example, would enable free-text symptom text to be resurfaced as a list of existing AEs to be easily rescored at future time points.

Collecting the most bothersome AEs (Figure 3B) has similarities to some existing PROMs that measure the most bothersome symptoms (MBSs). For example, MBS has been shown to be a useful patient-centric measure of migraine symptoms [26,27] and is referred to in the patient-focused drug development guidance published by the FDA [28]. Challenges with collecting an MBS include how to pool data in the statistical analysis and different symptoms becoming the most bothersome over time. However, these challenges may be less relevant when collecting the set of most bothersome AEs to understand the dose-toxicity relationship in early phase cancer trials.

### Other Recommended PROMs

**Figure 4.**
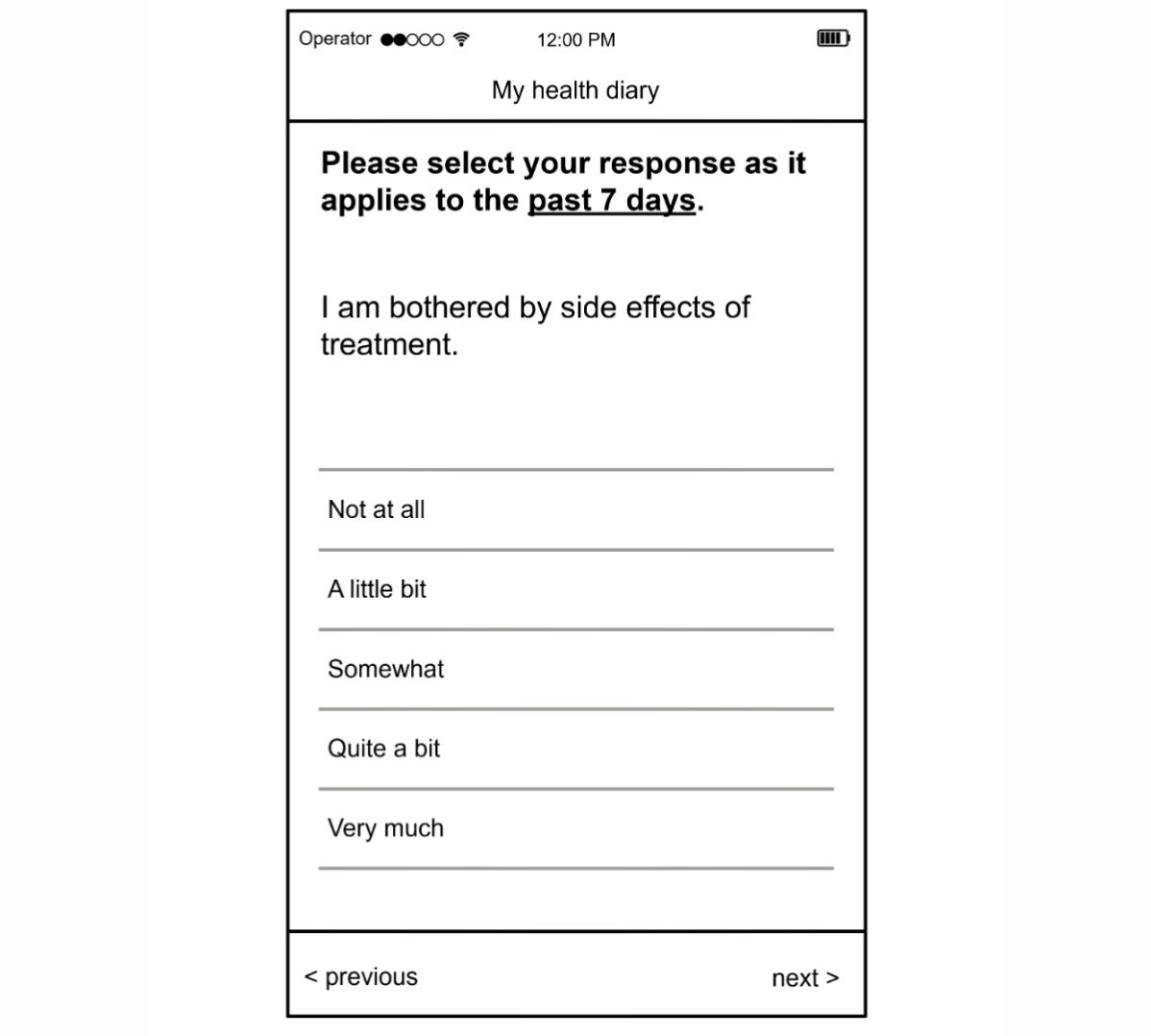
Functional Assessment of Cancer Therapy–Item GP5 (FACT GP5). An example of a single-item measure of the impact of adverse events. FACT GP5 is reproduced with permission, copyright of Dr David Cella, and licensed by FACIT.org [18].

The Friends of Cancer white paper [8] and FDA guidance on PROs in cancer trials [21] both recommend that in addition to the collection and scoring of individual AEs, an overall measure of the AE impact is included, along with measures to assess the impact of treatment on physical function and role function, although the FDA guidance is more focused toward use in confirmatory trials. A single item to score the overall impact of AEs, such as the FACT GP5 [18] (Figure 4) or Q168 of the EORTC item bank [19], is important as it enables the patient to account for the impact of any AEs not covered by PRO-CTCAE administration and to attach greater importance to the combined impact of multiple low-level AEs. This understanding may be particularly important in assessing the impact of newer treatments taken over longer periods. The FDA has identified measures of physical function and role function they consider suitable, including the EORTC QLQ-C30 physical function and role function subscales [20] and the Patient-Reported Outcomes Measurement Information System (PROMIS) physical function scale [29].

### Mitigating Barriers to PROM Adoption in Early Phase Trials

As described earlier, barriers to the adoption of PROMs in early phase cancer trials include a lack of guidance regarding PROM selection, concerns relating to dealing with missing PRO data, overburdening site staff and patients, handling patient and data queries [23], and low power associated with small cohorts. We address PROM selection in the discussion above. Dealing with missing data is always an important consideration in clinical research, as different missing data approaches rely on assumptions that, if violated, can lead to biased estimates. Although researchers in early phase trials may use less formal approaches to interpreting the data and determining the optimal dose, it will remain important to consider the impact of missing data using a variety of sensitivity evaluations.

In terms of patient burden, the UK National Cancer Research Institute Consumer Forum survey indicated that most patients and carers affected by cancer and involved in research activities (n=57) were willing to spend up to 15 minutes per day completing PROMs [23]. This time duration seems high for frequent collection but perhaps reflects the value that patients see in communicating this data to their treating physician. The measures we have discussed above typically use a 7-day recall period, and it is, therefore, most likely that a weekly completion schedule would be recommended. Recall bias using the weekly recall periods associated with these validated measures is unlikely to be a concern, but ensuring completion times do not overburden patients with the debilitating effects of the disease and treatment is an important consideration. Median per-item completion rates of PROMs commonly used in oncology trials have been reported as 6‐14 seconds [30], which suggests that a weekly PROM assessment of, for example, the PRO-CTCAE implemented using the approach outlined in Figure 3A, an overall AE impact item, and the physical function and role function subscales of the QLQ-C30 (items 5 and 2, respectively) might translate to an average completion time of less than 5 minutes per week. This seems to be a feasible assessment strategy, and ensuring a flexible completion window across more than 1 day may drive higher completion rates.

The remaining barriers cited may be mitigated by electronic collection of PRO data, for example, by using an ePROs smartphone app. The burden on site staff and patients during busy clinic visits can be mitigated by enabling at-home completion, and electronic tools can eliminate data queries by prohibiting ambiguous or invalid entries. The easy implementation of longer lists of items using branching logic to speed completion is only practical using an electronic approach. Further, the use of ePRO solutions can also lead to reduced missing data through alarms, reminders, and remote monitoring to drive on-time completion.

Electronic collection of PRO data may be perceived as a significant additional cost relative to the smaller numbers of patients involved in early phase studies, but this should be considered in the context of the value of the data. The more frequent assessment schedules and the nature of the measures implemented drive the use of electronic solutions. In the context of the increased expense of studying more patients in early phase trials due to the need to better characterize dose response, the use of ePROs to drive more accurate, reliable data may lead to accurate decision-making using relatively smaller sample sizes and offset the cost of ePROs many times over.

Smaller sample sizes associated with early phase studies may limit the robust characterization of the dose-response relationship, but this limitation is not unique to PRO data and also applies to other measures of efficacy and tolerability that inform dose selection. A thoughtful approach is required to balance the cost of increased sample size with the statistical robustness of dose-response characterization. Supplementary qualitative data collected using in-trial patient interviews may be a valuable addition to understanding the AEs experienced by the patients and aid the interpretation of PROM data when limited by small sample sizes.

## Conclusion

There is growing interest in more completely quantifying the dose-response relationship to inform optimal dose determination for new oncological treatments. PROs play a vital role in understanding dose tolerability profiles, especially as treatment-related AEs tend to be underreported and underscored by physicians. While AE profiles are less understood in early phase drug development, this should not prevent the capturing of this data to inform dose selection decisions as early as the first in human study using some of the approaches discussed in this paper.

Of course, we lack experience in interpreting PRO tolerability data from such early studies and need to remember that we may not have adequately defined the patient population at this early stage, and so the clinical interpretation of dose-response relationships associated with the PROs and other efficacy and tolerability data needs to be interpreted with this in mind.

With newer targeted therapeutics, there is a need to learn much more about safety and tolerability across a wide range of doses, and the current dose-finding models focusing on a single “optimal” dose may no longer work. A fundamental element of the decision-making process for determining safety and tolerability currently missing is the patient experience. It has been assumed that the physician, through patient interaction and AE reporting, can provide a sufficient reflection of the patient experience, but the evidence demonstrates that this is not reliable. Some important symptoms for patients are missed completely. The severity of other symptoms is underreported. Further, with newer targeted agents, AEs may accumulate over time, and the chronic nature or combination of events may make a dose become intolerable for the patient later. If the patient’s perspective is not considered, there is a risk of selecting dose groups that are too high, leading to reduced compliance. It is, therefore, necessary to build a PROM assessment strategy for early phase trials that combines elements of well-established scales to assess safety and tolerability in a package that is practical and not burdensome, yielding vital data to support decision-making as the trial progresses. Maximizing the value of the early patient experience is ethically appropriate and feasible, and drives efficiency in development programs and patient exposure.

## Abbreviations

AE: adverse event
CTCAE: Common Terminology Criteria for Adverse Events
EORTC: European Organisation for Research and Treatment of Cancer
EORTC QLQ-C30: EORTC Quality of Life Questionnaire–Core Questionnaire
ePRO: electronic patient-reported outcome
FACT GP5: Functional Assessment of Cancer Therapy–Item GP5
FDA: Food and Drug Administration
MBS: most bothersome symptom
PRO: patient-reported outcome
PRO-CTCAE: Patient-Reported Outcomes version of the Common Terminology Criteria for Adverse Events
PROM: patient-reported outcome measure
PROMIS: Patient-Reported Outcomes Measurement Information System
